# Learning and deskilling effects of artificial intelligence in colonoscopy among endoscopists with different levels of experience: a pragmatic, prospective trial

**DOI:** 10.1055/a-2858-7084

**Published:** 2026-06-03

**Authors:** Tom Andre Pedersen, Yuichi Mori, Edoardo Botteri, Trond Engjom, Birgitte Seip, Georg Gjorgji Dimcevski, Roald Flesland Havre

**Affiliations:** 1 Department of Medicine 60498 Haukeland University HospitalBergenNorway; 2 Department of MedicineHaraldsplass Deaconess HospitalBergenNorway; 3 Bergen Research Group for Advanced Gastrointestinal Endoscopy (BRAGE), Department of Clinical Medicine (K1) 1658 University of BergenBergenNorway; 4 Clinical Effectiveness Research Group 6305 University of OsloOsloNorway; 5 Department of Colorectal Cancer ScreeningCancer Registry of Norway, Norwegian Institute of Public HealthOsloNorway; 6 Department of ResearchCancer Registry of Norway, Norwegian Institute of Public HealthOsloNorway; 7 Department of Clinical Medicine (K1)University of BergenBergenNorway; 8 Department of Medicine 60512 Vestfold Hospital TrustTønsbergNorway; 9 Kanalspesialistene ASBergenNorway

## Abstract

**Background:**

The effect of computer-aided detection (CADe) on performance of endoscopists with different experience levels is not well understood. This study assessed whether CADe use promotes learning or deskilling.

**Methods:**

We performed a prospective, multicenter, registry-based, pragmatic clinical trial. The primary end point was change in the proportion of colonoscopies with detection of at least one polyp ≥5 mm (PDR-5). Each endoscopist performed colonoscopies in three successive phases: 1) before CADe exposure, 2) during CADe use, and 3) after CADe removal. To assess the impact of CADe on PDR-5, we compared phase 1 with phases 2 and 3, adjusting for patient and endoscopist characteristics and endoscopy performance measures. Generalized linear mixed models were used and presented according to endoscopist experience.

**Results:**

13 endoscopists (7 inexperienced, 6 experienced) performed 5013 colonoscopies; median patient age was 59 years (interquartile range 44–71) and 2703 (53.9%) were women. Patient sex and age were similar across the two experience groups, whereas indications differed (e.g. more inflammatory bowel disease in the inexperienced group). CADe temporarily increased PDR-5 among inexperienced endoscopists from phase 1 to 2 (31.9% [216/678] to 39.5% [257/651]; odds ratio [OR] 1.43, 95%CI 1.11–1.84). There was no significant change between phases 1 and 3 (31.9% [216/678] to 36.3% [173/476]; OR 1.03, 95%CI 0.79–1.34). There was no significant CADe effect on PDR-5 among experienced endoscopists between phases.

**Conclusions:**

PDR-5 of inexperienced endoscopists increased when CADe was used. In non-CADe-assisted colonoscopy, no upskilling or deskilling was observed following a period of CADe exposure.

## Abstract (de)

**Graphical Abstract:**

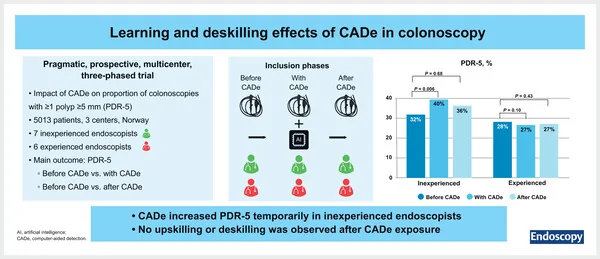

## Introduction

An increased adenoma detection rate (ADR) reduces the risk of post-colonoscopy colorectal cancer (CRC) and death [Bibr LI_LiteratureBookmark_1] [Bibr LI_LiteratureBookmark_2] [Bibr LI_LiteratureBookmark_3] . High-quality colonoscopies are vital for preventing and treating CRC. In recent years, artificial intelligence (AI) has been introduced into colonoscopy for polyp detection (i.e. computer-aided detection [CADe]), which is expected to increase ADR according to randomized trials [Bibr LI_LiteratureBookmark_4] [Bibr LI_LiteratureBookmark_5] [Bibr LI_LiteratureBookmark_6] [Bibr LI_LiteratureBookmark_7] . However, in contrast to such promising initial results, more recent real-world studies have questioned the effect of CADe [Bibr LI_LiteratureBookmark_8] [Bibr LI_LiteratureBookmark_9] . Furthermore, a newly published article even showed a deskilling effect after the introduction of CADe for polyp detection [Bibr LI_LiteratureBookmark_10] . Nevertheless, there is still a scarcity of evidence on whether the use of CADe contributes to the improvement or deskilling of endoscopists over time. Given the unresolved questions surrounding human–AI interaction in endoscopy, the European Society of Gastrointestinal Endoscopy (ESGE) recently published a curriculum aimed at ensuring the safe and effective use of AI in this field [Bibr LI_LiteratureBookmark_11] .

To address the important question of learning effects from CADe, we evaluated how CADe use affects the polyp detection rate (PDR)-5, defined as the proportion of colonoscopies with detection of at least one polyp ≥5 mm in size; PDR-5 is a surrogate marker for ADR in Gastronet, the Norwegian national endoscopy quality registry [Bibr LI_LiteratureBookmark_12] . Gastronet utilizes PDR-5 as a convenient quality indicator for colonoscopy, as it correlates with ADR and does not require histological confirmation.

In the present study, we aimed to compare PDR-5 of endoscopists in training across three consecutive phases: 1) before CADe exposure, 2) during CADe use, and 3) after CADe removal. We also investigated the same measures for experienced endoscopists.

## Methods

### Design and patients

We performed a prospective, multicenter, registry-based, pragmatic clinical trial from November 2021 to June 2025, including patients who underwent colonoscopy at three Norwegian outpatient clinics: a university clinic (Haukeland University Hospital), a local hospital (Haraldsplass Deaconess Hospital), and a high-volume outpatient endoscopy practice (Kanalspesialistene).

Each endoscopist aimed to perform at least 100 colonoscopies in each of three successive phases: phase 1 – before CADe exposure; phase 2– during CADe use; and phase 3 – after CADe removal. Each endoscopist completed the three consecutive phases in a fixed order, serving as their own controls. We divided the endoscopists into two groups: inexperienced and experienced. We defined “inexperienced” as an endoscopist who had performed between 100 and 1000 colonoscopies, and “experienced” as someone who had performed more than 1000 colonoscopies in their career before the study began. To perform a technically adequate procedure, the participating endoscopists had to have performed at least 100 colonoscopies in their career before inclusion began. The term “pragmatic” refers to a trial designed to assess clinical effectiveness in real-world settings, rather than measuring efficacy in an ideal or controlled environment. This type of trial aims to address the variability found in clinical practices and among patients [Bibr LI_LiteratureBookmark_7] .

The colonoscopies were performed as part of the regular outpatient clinic with registration in our national endoscopy quality registry, Gastronet. To evaluate how CADe affects real-world clinical colonoscopy, we excluded patients with a positive fecal immunochemical test in the Norwegian CRC screening program. Screening was not performed in all participating centers, and much higher ADRs are achieved in screening colonoscopies than those performed for other indications, which could represent a possible selection bias in our study population [Bibr LI_LiteratureBookmark_13] . Gastronet collects information from both endoscopists and patients, providing data such as cecal intubation rate, bowel cleansing (as measured by the Boston Bowel Preparation Scale [BBPS]), withdrawal time, PDR-5, indication, use of sedation/analgesia, and patient-reported outcome measures, including pain grading and information provided before and after the procedure. Adequate bowel cleansing in Gastronet is defined as BBPS ≥6 with no segment score (right, transverse, left colon) being <2. Gastronet uses PDR-5 as a surrogate marker for ADR. The endoscopist estimates polyp size using a biopsy forceps or polypectomy snare as a reference. PDR-5 is readily available, not dependent on histology, and encompasses both adenomas and serrated polyps. A meta-analysis by Niv et al. examined the correlation between ADR and PDR (polyps of all sizes) and found a conversion factor of 0.68 to calculate ADR from PDR [Bibr LI_LiteratureBookmark_14] . In Gastronet, the registered withdrawal time is the time spent inspecting the mucosa during withdrawal of the colonoscope from the cecum to the anal canal, rounded to the nearest whole minute, also including polypectomies and/or biopsies if performed [Bibr LI_LiteratureBookmark_15] . The cases classified as CRC family/“screening” refer to surveillance colonoscopies in families with CRC or colonoscopies that are not part of the official CRC screening program.

We used the CADe system GI Genius (Medtronic, Minneapolis, Minnesota USA). Cosmo Intelligent Medical Devices (Dublin, Ireland) developed this CADe system, and Medtronic distributes it. GI Genius is compatible with most endoscopy systems. Technical details have been described previously [Bibr LI_LiteratureBookmark_16] . We used CADe software version 2 for this study. Computer-aided diagnosis was not available to the endoscopists. Before the study began, the endoscopists underwent standard training on polyp characterization and the use of CADe. They were advised to activate the AI system when withdrawing the colonoscope from the cecum. The CADe system remained active throughout the entire inspection phase during the withdrawal of the colonoscope. The endoscopists used Olympus high-definition video processors and colonoscopes (Olympus Medical Systems, Tokyo, Japan).

### Study assumption and end points

We hypothesized that real-time use of CADe during phase 2 would increase PDR-5 in both inexperienced and experienced endoscopists. We also hypothesized that the increased PDR-5 would persist even after CADe was removed in phase 3.

Our primary end points were changes in PDR-5 between phases 1 and 2, and between phases 1 and 3, for both inexperienced and experienced endoscopists, respectively.

### Ethical considerations

Gastronet uses an anonymous registration number linked to the patient, the endoscopist, and the center. Information in Gastronet is collected in accordance with the Norwegian Directorate of Health’s exemption from the duty of confidentiality. The Norwegian Data Protection Authority licenses the register. The concession does not require patient consent for registration; however, patients have the right to opt out of registration and are informed of the procedure in writing. The participating endoscopists provided informed consent for their registered data in Gastronet to be used. The endoscopists underwent a standard course that included adherence to the study protocol and instruction on data registration. The Regional Committee for Medical and Health Research Ethics (REC) in Western Norway approved the study (REC identification number 275068).

### Power analysis

We anticipated a PDR-5 of 30% before CADe use and an improvement to 35% during CADe use (5% absolute improvement) for both experience groups. This expected 5% absolute improvement was based on publications reporting ADR improvement with CADe available at the time of study planning in 2021 [Bibr LI_LiteratureBookmark_17] [Bibr LI_LiteratureBookmark_18] [Bibr LI_LiteratureBookmark_19] [Bibr LI_LiteratureBookmark_20] . To detect a 5% absolute increase in PDR-5 with a two-sided test, an alpha of 0.05, and a power of 80%, we required 688 colonoscopies in phase 1 and 688 in phase 2 for both inexperienced and experienced groups. We chose to include the same number of colonoscopies in phase 3. This resulted in 4128 colonoscopies. Because some newer studies failed to demonstrate statistical significance, we increased the sample size to 5000 colonoscopies [Bibr LI_LiteratureBookmark_8] . With this adjustment, the study power increased to 87%.

### Statistical analysis

Continuous variables were presented as medians with interquartile ranges (IQRs) or as means with SDs. Categorical variables were presented as frequencies and percentages. Pearson’s chi-squared test was used to compare categorical variables between groups, and Student’s *t* test was used to compare continuous variables between groups. Generalized linear mixed models were used to estimate the association between explanatory variables and the dependent variable, PDR-5. The following explanatory variables were entered as fixed effects: CADe phase, patient sex and age, colonoscopy indication, BBPS, cecal intubation rate, sedation/analgesia, inclusion time from first colonoscopy, and endoscopists’ experience. The endoscopist was included as a random effect (subject variable) to account for clustering of procedures within endoscopists. We also performed sensitivity analyses with center as a fixed effect, but the estimated effects of all covariates, as well as the overall conclusions, were not materially different from those that did not include center. For this reason, we did not include center in the model. The explanatory variables with *P* values of <0.1 in the univariable analyses were included in the multivariable analyses. Withdrawal time was not included in the generalized linear mixed models because it was only collected for patients without biopsies or polypectomies. Pain was also excluded, as only 46.7% (2341/5013) of patients returned the patient-reported outcome measures after endoscopy. We reported odds ratios (ORs) and 95%CIs. Statistical significance was defined as a *P* value of <0.05 using two-sided tests. Data were analyzed using IBM SPSS Statistics Version 30 (IBM Corp., Armonk, New York, USA).

## Results

We included 5034 colonoscopies from 13 endoscopists who participated in all three phases ( [Fig FI_Ref230163972] ). After excluding 14 colonoscopies due to insufficient demographic data and 7 due to two colonoscopies being performed in the same patient, we analyzed 5013 colonoscopies performed by 7 inexperienced and 6 experienced endoscopists.

**Figure FI_Ref230163972:**
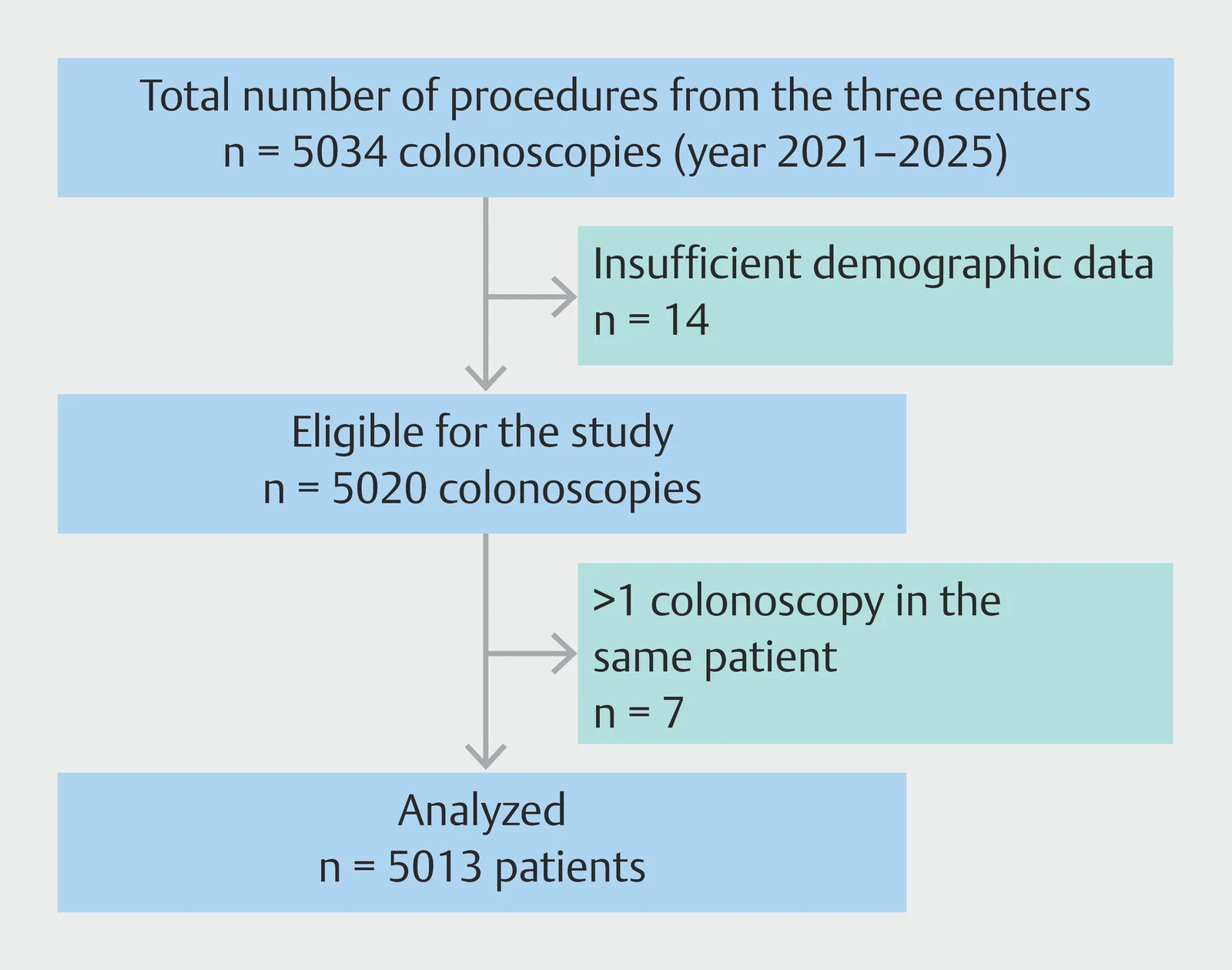
Patient inclusion.

The 13 endoscopists comprised 12 physicians and 1 surgeon. At the beginning of the study, the inexperienced endoscopist group had a mean age of 41.1 years, whereas the experienced group had a mean age of 50.7 years.

Mean inclusion times from the first colonoscopy among the inexperienced vs. experienced groups were 6.8 vs. 2.2 months in phase 1, 17.7 vs. 7.6 months in phase 2, and 23.8 vs. 11.8 months in phase 3, respectively.

Patient background characteristics across the three study phases were generally similar with respect to age and sex, in both endoscopist experience groups. However, the indications for colonoscopy differed slightly, with, for example, a higher proportion of symptomatic patients in phase 3. The inexperienced group had more patients with inflammatory bowel disease (IBD) in all phases, and the experienced group had more female patients in phases 1 and 2 ( [Table TB_Ref230165405] ; see also **Table 1s** in the online-only Supplementary material). [Table TB_Ref230165400] shows the number of colonoscopies performed per endoscopist and the PDR-5 in the three phases, respectively. Most endoscopists reached at least 100 colonoscopies in phases 1 and 2, but some inexperienced endoscopists performed only a few colonoscopies in phase 3. **Table 1s** also shows the quality indicators in the three phases for the different experience levels.

**Table TB_Ref230165405:** Patient and endoscopist characteristics, and quality indicators.

	Total	Phase 1	Phase 2 (CADe)	Phase 3	*P* value
Patients, n (%)	5013 (100)				
Inexperienced		678 (100)	651 (100)	476 (100)	0.33
Experienced		1075 (100)	1088 (100)	1045 (100)	0.46
Women	2703 (53.9)				
Inexperienced		343 (50.6)	330 (50.7)	260 (54.6)	
Experienced		609 (56.7)	596 (54.8)	565 (54.1)	
Men	2310 (46.1)				
Inexperienced		335 (49.4)	321 (49.3)	216 (45.4)	
Experienced		466 (43.3)	492 (45.2)	480 (45.9)	
Age, median (IQR), years	59 (44–71)				
Inexperienced		56 (40–71)	54 (39–69)	61 (43–73)	0.10
Experienced		59 (45–70)	61 (46.25–71.75)	59 (45–70)	0.08
Indication, n (%)					
Inexperienced					**<0.001**
Experienced					**0.03**
Symptoms	3015 (60.1)				
Inexperienced		353 (52.1)	333 (51.2)	300 (63.0)	
Experienced		658 (61.2)	660 (60.7)	711 (68.0)	
Surveillance (polyp, CRC)	580 (11.6)				
Inexperienced		71 (10.5)	107 (16.4)	69 (14.5)	
Experienced		123 (11.4)	126 (11.6)	84 (8.0)	
IBD	664 (13.2)				
Inexperienced		175 (25.8)	163 (25.0)	72 (15.1)	
Experienced		89 (8.3)	87 (8.0)	78 (7.5)	
CRC family/“screening” ^1^	262 (5.2)				
Inexperienced		42 (6.2)	8 (1.2)	11 (2.3)	
Experienced		78 (7.3)	69 (6.3)	54 (5.2)	
Other	458 (9.1)				
Inexperienced		33 (4.9)	35 (5.4)	23 (4.8)	
Experienced		118 (11.0)	138 (12.7)	111 (10.6)	
Missing	34 (0.7)				
Inexperienced		4 (0.6)	5 (0.8)	1 (0.2)	
Experienced		9 (0.8)	8 (0.7)	7 (0.7)	
**Bold***P* values are significant. CADe, computer-aided detection; CRC, colorectal cancer; IBD, inflammatory bowel disease; IQR, interquartile range; PDR-5, proportion of colonoscopies with ≥1 polyp ≥5 mm in size. ^1^ “Screening” refers to surveillance colonoscopies in families with CRC or colonoscopies that are not part of the official CRC screening program.					

**Table TB_Ref230165400:** Proportion of colonoscopies with at least one polyp ≥5 mm in size (PDR-5) per endoscopist in each phase.

	PDR-5		
	Phase 1, n/N (%)	Phase 2, n/N (%)	Phase 3, n/N (%)
Experienced endoscopists (n = 6)			
	78/216 (36.1)	73/213 (34.3)	74/260 (28.5)
	31/83 (37.3)	36/103 (35.0)	41/107 (38.3)
	11/95 (11.6)	13/100 (13.0)	11/117 (9.4)
	93/325 (28.6)	64/312 (20.5)	64/255 (25.1)
	13/91 (14.3)	22/100 (22.0)	13/47 (27.7)
	78/265 (29.4)	83/260 (31.9)	81/259 (31.3)
Total	304/1075 (28.3)	291/1088 (26.7)	284/1045 (27.2)
Inexperienced endoscopists (n = 7)			
	22/96 (22.9)	28/100 (28.0)	3/17 (17.6)
	48/104 (46.2)	53/107 (49.5)	35/94 (37.2)
	38/106 (35.8)	48/101 (47.5)	50/112 (44.6)
	20/91 (22.0)	16/69 (23.2)	3/25 (12.0)
	30/98 (30.6)	30/92 (32.6)	25/82 (30.5)
	26/71 (36.6)	47/100 (47.0)	53/132 (40.2)
	32/112 (28.6)	35/82 (42.7)	4/14 (28.6)
Total	216/678 (31.9)	257/651 (39.5)	173/476 (36.3)
All endoscopists	520/1753 (29.7)	548/1739 (31.5)	457/1521 (30.0)

### Univariable and multivariable analyses of PDR-5

[Table TB_Ref230165359] and [Table TB_Ref230165807] show the results of the univariable and multivariable analyses assessing factors associated with PDR-5. Separate analyses were performed for phase 1 vs. phase 2 ( [Table TB_Ref230165359] **, Table 2s** ) and phase 1 vs. phase 3 ( [Table TB_Ref230165807] **, Table 3s** ) to evaluate the effect of CADe (phase 2) and whether this effect persisted after its use (phase 3). The effects of CADe across study phases and endoscopist experience levels are illustrated in [Fig FI_Ref230163966] .

**Figure FI_Ref230163966:**
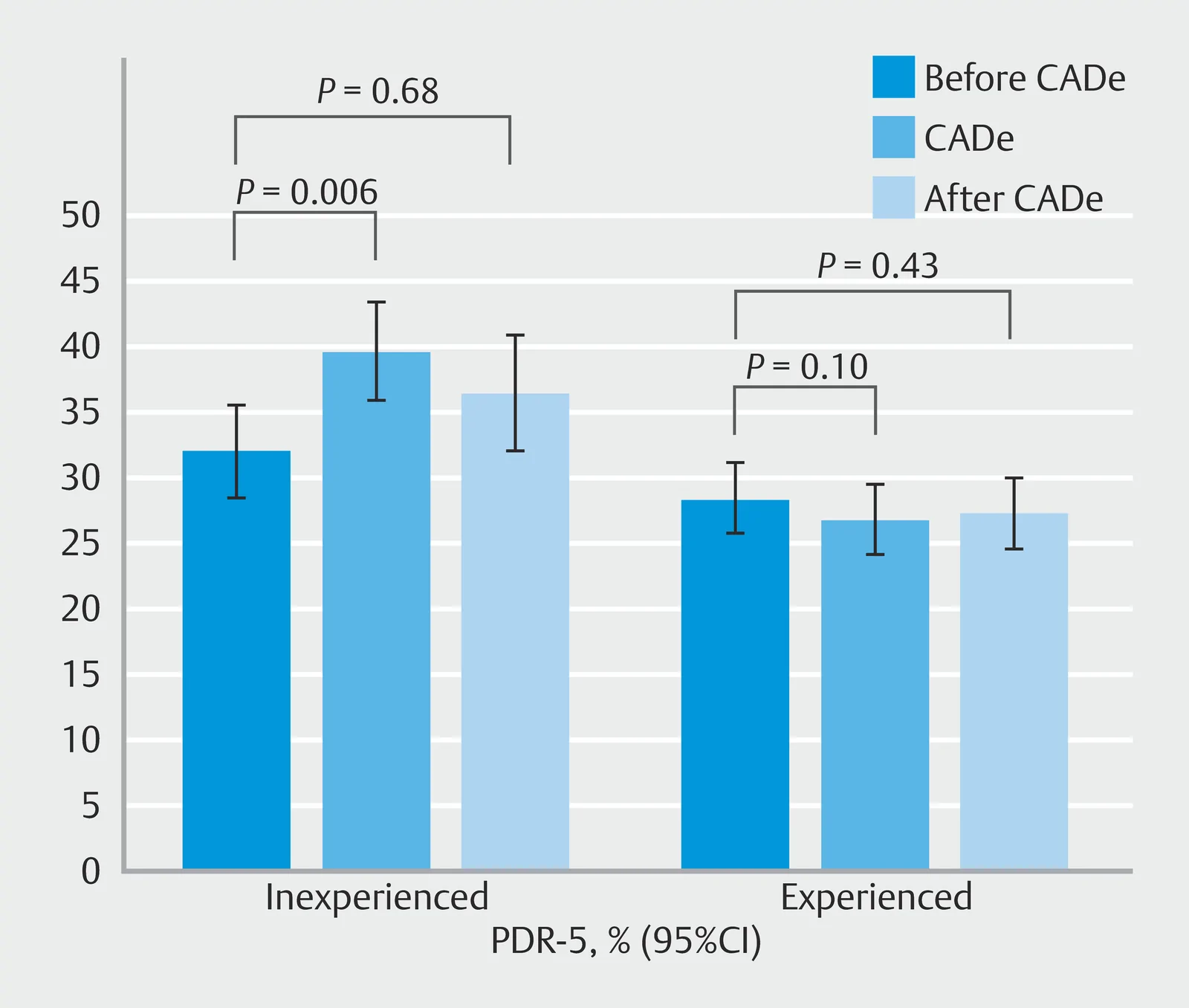
Proportion (with 95%CI) of colonoscopies with at least one polyp ≥5 mm in size (PDR-5). Results from multivariable analyses.

**Table TB_Ref230165359:** Proportion of colonoscopies with at least one polyp ≥5 mm in size (PDR-5) in phases 1 and 2.

Variable/category	PDR-5, n/N (%)	Univariable analysis, OR (95%CI)	Multivariable analysis, OR (95%CI)
Phase			
1 (before CADe) (ref.)			
All	520/1753 (29.7)	–	–
Inexperienced	216/678 (31.9)	–	–
Experienced	304/1075 (28.3)	–	–
2 (CADe)			
All	548/1739 (31.5)	1.09 (0.94–1.26)	–
Inexperienced	257/651 (39.5)	1.37 (1.09–1.72) ^1^	1.43 (1.11–1.84) ^1^
Experienced	291/1088 (26.7)	0.92 (0.76–1.12)	–
Sex			
Women (ref.)			
All	515/1878 (27.4)	–	–
Inexperienced	207/673 (30.8)	–	–
Experienced	308/1205 (25.6)	–	–
Men			
All	553/1614 (34.3)	1.39 (1.20–1.61) ^1^	1.33 (1.12–1.56) ^1^
Inexperienced	266/656 (40.5)	1.56 (1.24–1.97) ^1^	1.54 (1.20–1.98) ^1^
Experienced	287/958 (30.0)	1.28 (1.05–1.55) ^1^	1.14 (0.92–1.41)
Age			
5-year increase			
All	1068/3492 (30.6)	1.20 (1.17–1.23) ^1^	1.17 (1.14–1.20) ^1^
Inexperienced	473/1329 (35.6)	1.22 (1.18–1.27) ^1^	1.19 (1.15–1.24) ^1^
Experienced	595/2163 (27.5)	1.18 (1.14–1.22) ^1^	1.15 (1.11–1.19) ^1^
Indication			
Symptoms (ref.)			
All	595/2004 (29.7)	–	–
Inexperienced	240/686 (35.0)	–	–
Experienced	355/1318 (26.9)	–	–
Surveillance (polyp, CRC)			
All	241/427 (56.4)	3.22 (2.58–4.02) ^1^	2.49 (1.97–3.14) ^1^
Inexperienced	117/178 (65.7)	3.73 (2.62–5.30) ^1^	2.69 (1.86–3.89) ^1^
Experienced	124/249 (49.8)	2.86 (2.15–3.82) ^1^	2.32 (1.71–3.13) ^1^
IBD			
All	82/514 (16.0)	0.42 (0.32–0.55) ^1^	0.52 (0.39–0.70) ^1^
Inexperienced	71/338 (21.0)	0.52 (0.38–0.71) ^1^	0.68 (0.49–0.95) ^1^
Experienced	11/176 (6.3)	0.21 (0.11–0.40) ^1^	0.25 (0.13–0.48) ^1^
CRC family/“screening” ^3^			
All	45/197 (22.8)	0.84 (0.58–1.19)	0.83 (0.58–1.20)
Inexperienced	18/50 (36.0)	1.34 (0.72–2.47)	1.36 (0.72–2.58)
Experienced	27/147 (18.4)	0.66 (0.42–1.02) ^2^	0.70 (0.45–1.10)
Other			
All	98/324 (30.2)	1.21 (0.93–1.58)	1.02 (0.78–1.35)
Inexperienced	25/68 (36.8)	1.26 (0.74–2.15)	1.03 (0.59–1.79)
Experienced	73/256 (28.5)	1.16 (0.85–1.58)	1.00 (0.73–1.38)
Missing			
All	7/26 (26.9)	0.80 (0.32–1.95)	0.83 (0.33–2.12)
Inexperienced	2/9 (22.2)	0.60 (0.12–2.97)	0.58 (0.10–3.28)
Experienced	5/17 (29.4)	0.85 (0.29–2.54)	0.88 (0.29–2.71)
The univariable analyses were based on generalized linear mixed models, with the endoscopist as a clustering random effect. CADe, computer-aided detection; CRC, colorectal cancer; IBD, inflammatory bowel disease; OR, odds ratio. All univariable analyses with *P * < 0.1 were included in the multivariable analyses. ^1^ *P * < 0.05. ^2^ *P * < 0.10. ^3^ “Screening” refers to surveillance colonoscopies in families with CRC or colonoscopies that are not part of the official CRC screening program.			

**Table TB_Ref230165807:** Proportion of colonoscopies with at least one polyp ≥5 mm in size (PDR-5) in phases 1 and 3.

Variable/category	PDR-5, n/N (%)	Univariable analysis, OR (95%CI)	Multivariable analysis, OR (95%CI)
Phase			
1 (before CADe) (ref.)			
All	520/1753 (29.7)	–	–
Inexperienced	216/678 (31.9)	–	–
Experienced	304/1075 (28.3)	–	–
3 (after CADe)			
All	457/1521 (30.0)	0.95 (0.82–1.11)	–
Inexperienced	173/476 (36.3)	1.03 (0.79–1.34)	–
Experienced	284/1045 (27.2)	0.93 (0.76–1.12)	–
Sex			
Women (ref.)			
All	494/1777 (27.8)	–	–
Inexperienced	185/603 (30.7)	–	–
Experienced	309/1174 (26.3)	–	–
Men			
All	483/1497 (32.3)	1.25 (1.08–1.46) ^1^	1.23 (1.04–1.45) ^1^
Inexperienced	204/551 (37.0)	1.35 (1.06–1.73) ^1^	1.35 (1.04–1.76) ^1^
Experienced	279/946 (29.5)	1.19 (0.98–1.45) ^2^	1.12 (0.90–1.38)
Age			
5-year increase			
All	977/3274 (29.8)	1.20 (1.17–1.23) ^1^	1.17 (1.14–1.20) ^1^
Inexperienced	389/1154 (33.7)	1.20 (1.16–1.25) ^1^	1.18 (1.13–1.22) ^1^
Experienced	588/2120 (27.7)	1.19 (1.15–1.23) ^1^	1.17 (1.13–1.21) ^1^
Indication			
Symptoms (ref.)			
All	574/2022 (28.4)	–	–
Inexperienced	217/653 (33.2)	–	–
Experienced	357/1369 (26.1)	–	–
Surveillance (polyp, CRC)			
All	190/347 (54.8)	3.07 (2.42–3.90) ^1^	2.37 (1.85–3.03) ^1^
Inexperienced	88/140 (62.9)	3.44 (2.34–5.04) ^1^	2.74 (1.84–4.08) ^1^
Experienced	102/207 (49.3)	2.84 (2.09–3.87) ^1^	2.18 (1.58–2.99) ^1^
IBD			
All	62/414 (15.0)	0.44 (0.33–0.60) ^1^	0.56 (0.41–0.76) ^1^
Inexperienced	47/247 (19.0)	0.50 (0.35–0.72) ^1^	0.66 (0.45–0.98) ^1^
Experienced	15/167 (9.0)	0.33 (0.18–0.58) ^1^	0.38 (0.21–0.69) ^1^
CRC family/“screening” ^3^			
All	45/185 (24.3)	0.92 (0.64–1.32)	0.91 (0.63–1.31)
Inexperienced	17/53 (32.1)	1.20 (0.65–2.22)	1.19 (0.63–2.23)
Experienced	28/132 (21.2)	0.79 (0.51–1.24)	0.80 (0.51–1.26)
Other			
All	100/285 (35.1)	1.51 (1.15–1.98) ^1^	1.29 (0.97–1.70)
Inexperienced	20/56 (35.7)	1.25 (0.70–2.22)	1.04 (0.57–1.90)
Experienced	80/229 (34.9)	1.57 (1.56–2.14) ^1^	1.37 (1.00–1.88) ^2^
Missing			
All	6/21 (28.6)	0.97 (0.37–2.59)	0.93 (0.34–2.55)
Inexperienced	0/5 (0.0)	–	–
Experienced	6/16 (37.5)	1.41 (0.49–4.07)	1.26 (0.42–3.73)
The univariable analyses were based on generalized linear mixed models, with the endoscopist as a clustering random effect. CADe, computer-aided detection; CRC, colorectal cancer; IBD, inflammatory bowel disease; OR, odds ratio. All univariable analyses with *P * < 0.1 were included in the multivariable analyses. ^1^ *P * < 0.05. ^2^ *P * < 0.10. ^3^ “Screening” refers to surveillance colonoscopies in families with CRC or colonoscopies that are not part of the official CRC screening program.			

CADe use was significantly associated with an increased PDR-5 among inexperienced endoscopists ( [Table TB_Ref230165359] ) (OR 1.43, 95%CI 1.11–1.84). CADe did not have a significant effect on experienced endoscopists. When comparing phase 1 (before CADe) with phase 3 (after CADe), there were no significant effects on PDR-5 overall or within the two endoscopist groups ( [Table TB_Ref230165807] ). Sex, age, and indication also significantly affected PDR-5 across all endoscopist groups. Time from first colonoscopy (divided into 3-month intervals) did not affect the PDR-5 significantly in any group ( **Table 2s** , **Table 3s** ).

### Endoscopist-level analysis

When looking at the inexperienced group, three deskilled, two unchanged, and two upskilled between phases 1 and 3. Similarly, among the experienced group, three deskilled and three upskilled ( [Table TB_Ref230165400] , [Fig FI_Ref230163961] ).

**Figure FI_Ref230163961:**
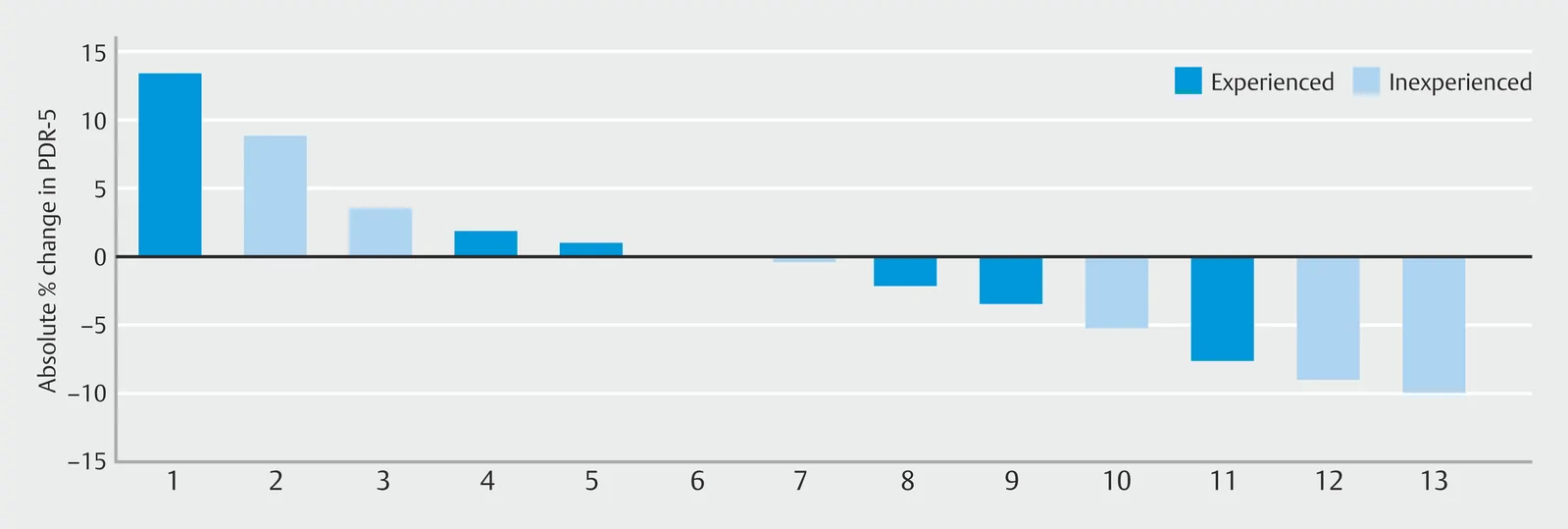
Change in proportion of colonoscopies with at least one polyp ≥5 mm in size (PDR-5) between phases 1 and 3 at the endoscopist level (n = 13).

## Discussion

We evaluated 5013 colonoscopies performed by seven inexperienced and six experienced endoscopists. An important finding with clinical impact was that CADe temporarily increased PDR-5 in inexperienced endoscopists, from 31.9% to 39.5% (OR 1.43, 95%CI 1.11–1.84) when CADe was used in phase 2. Another important finding was that we could not demonstrate upskilling or deskilling following CADe use. PDR-5 in non-CADe-assisted colonoscopies did not change significantly after CADe exposure, suggesting no obvious upskilling or deskilling (there was only a nonsignificant increase in PDR-5 after CAD exposure in the inexperienced group, from 31.9% to 36.3%). We observed no significant increase in PDR-5 during CAD use among experienced endoscopists. This differs from the study by Xu et al., which demonstrated an increased ADR regardless of experience [Bibr LI_LiteratureBookmark_21] . In fact, our study showed similar results for experienced endoscopists as the meta-analysis by Patel et al. and the study by Maas et al., which demonstrated no effect of CADe on ADR in a real-world setting among experienced endoscopists (nontrainees) [Bibr LI_LiteratureBookmark_8] [Bibr LI_LiteratureBookmark_9] .

The experienced endoscopists had a higher mean age than the inexperienced group (50.7 vs. 41.1 years), which may influence the effect of CADe through a conservatism bias in the human–AI interaction [Bibr LI_LiteratureBookmark_11] . We did not observe any effect of CADe among experienced endoscopists, suggesting that their skill in detecting polyps was not affected by CADe. Experienced endoscopists may underestimate, or at least do not overestimate, polyp size, whereas inexperienced endoscopists may be more prone to overestimation. The experienced endoscopists may also be less enthusiastic about a software tool intended to improve their performance, leading to an aversion to using it effectively. Algorithm aversion is addressed as a potential obstacle for human–AI interaction in the ESGE curriculum for safe and effective use of AI in endoscopy [Bibr LI_LiteratureBookmark_11] . Some endoscopists found the sound signal especially disturbing, but the box overlay did not provoke this aversion, though false-positive markings could be exhausting [Bibr LI_LiteratureBookmark_22] . Low false-positive rates are essential for CADe usability. GI Genius has to be actively turned on by the endoscopist. In the current study, two endoscopists reported having CADe active throughout the entire examination (including intubation of the colon), while the remaining endoscopists activated CADe on withdrawal from the cecum.

The finding of no significant effects of CADe on learning or deskilling among experienced endoscopists differs from the recent study by Budzyn et al., which found a significant deskilling effect among experienced endoscopists while they were exposed to CADe [Bibr LI_LiteratureBookmark_10] . A study by Troya et al. showed decreased eye travel distance when using CADe [Bibr LI_LiteratureBookmark_23] , which could explain why such deskilling happened in the study by Budzyn et al. However, there is a distinct difference in study design between our study and the Budzyn study. We evaluated the effect of exposure to CADe “after” CADe was removed, whereas Budzyn et al. investigated how endoscopists perform non-CADe-assisted colonoscopy “while” they were exposed to CADe in the other colonoscopies they performed during the same time period. Thus, it is considered that the effect of exposure to CADe will be stronger in the Budzyn study than in our study, suggesting that the deskilling effect may be a temporal phenomenon that disappears over time after CADe is removed.

Looking at the endoscopist-level analysis among the inexperienced endoscopists, three deskilled, two unchanged, and two upskilled between phases 1 and 3. Among the experienced group, three deskilled and three upskilled. This also implies that there has been neither an upskilling nor deskilling effect over time. However, it is challenging to draw such a conclusion based solely on the change in PDR-5 between phases 1 and 3, given that there is always a time effect in which endoscopists may naturally upskill or deskill over time. Mean inclusion time from the first colonoscopy among the inexperienced group was 6.8 months in phase 1, 17.7 months in phase 2, and 23.8 months in phase 3. Such long-term exposure to a medical procedure may play a role in skill development, which needs to be considered in analysis of upskilling and deskilling. This “time effect” problem applies to the recently published paper by Okumura et al. [Bibr LI_LiteratureBookmark_24] , which showed that, after CADe implementation, detection rates without CADe were maintained and did not decline over time. Among non-CADe colonoscopies, high detectors showed accelerated learning curves, indicating they maintained a higher ADR, whereas low detectors had no significant change in their learning curves. However, the inclusion period was quite long (3 years), compromising the study interpretation due to the possible “time effect” on learning.

In all phases, the inexperienced endoscopists had a higher PDR-5 than the experienced endoscopists. This may be related to inexperienced physicians overestimating polyp size or to their more meticulous mucosal evaluation than that of experienced physicians. The median withdrawal time across all phases was 10 minutes (IQR 8–16) for the inexperienced endoscopists and 9 minutes (IQR 7–11) for the experienced endoscopists. The withdrawal time must be evaluated with caution, however, as many examinations were excluded because of biopsies/therapy. Both experience groups had longer withdrawal times during the CADe phase. Other studies have shown similar results, with a slight increase in the withdrawal time using CADe [Bibr LI_LiteratureBookmark_4] . A study by Schult et al. showed that trainees detected more adenomas than consultants [Bibr LI_LiteratureBookmark_25] . Following an initial training period of 300 colonoscopies, ADR among trainees remained unchanged [Bibr LI_LiteratureBookmark_25] . This finding aligns with the study by Gianotti et al. [Bibr LI_LiteratureBookmark_26] , which demonstrated an increase in ADR after trainees performed more than 140 colonoscopies under supervision, and subsequently outperformed consultants. Thresholds of 325 colonoscopies in male patients and 539 colonoscopies in female patients were determined to be ideal for achieving adequate ADR. In the current study, we defined an experienced endoscopist as having performed ≥1000 colonoscopies during their career at the start of study inclusion. This may be an adequate cutoff when comparing our findings to those in these two previous studies [Bibr LI_LiteratureBookmark_25] [Bibr LI_LiteratureBookmark_26] . Our findings that inexperienced endoscopists outperform experienced endoscopists also align with these studies.

A strength of our study is the large number of colonoscopies performed by 13 endoscopists, both inexperienced and experienced. The more procedures included per endoscopist, the greater the reliability of the evaluation of changes in PDR-5. We increased the number of included endoscopies to approximately 5000 after the meta-analysis by Patel et al. showed no significant effect of CADe among experienced (nontrainee) endoscopists in a real-world setting [Bibr LI_LiteratureBookmark_8] . Another strength is the three-phase design, in which all endoscopists included colonoscopies in all phases, serving as their own controls. The colonoscopies were included at three different levels of outpatient care: university hospital, local hospital, and high-volume outpatient endoscopy practice. The design reflects a variety of endoscopy settings and experience levels, making this study broadly relevant for real-world endoscopy.

There are several limitations to this study. The most important is the use of PDR-5 as a quality indicator for lesion detection, rather than the gold standard ADR. A meta-analysis by Niv et al. found that a ratio of 0.68 can be used to estimate ADR from PDR for the individual endoscopist or a group of endoscopists before receiving formal results from the pathology department [Bibr LI_LiteratureBookmark_14] . PDR in colonoscopies is higher than PDR-5, suggesting that PDR-5 may have a ratio closer to 1 compared with PDR. PDR-5, being measured optically, may lead to over- or underestimation of polyp size, and studies have shown significant interphysician variation for estimating polyp size [Bibr LI_LiteratureBookmark_27] [Bibr LI_LiteratureBookmark_28] . To collect the large number of colonoscopies (approximately 5000), we decided to use PDR-5. We acknowledge that this quality indicator is less well-validated than ADR. Nevertheless, PDR-5 has been used for many years in Norway, and previous studies have adopted it [Bibr LI_LiteratureBookmark_12] [Bibr LI_LiteratureBookmark_29] . Another consideration was our aim to conduct a pragmatic study, in which endoscopists performed examinations according to their usual practice. Although polyps are sent to histology, we use PDR-5 as our quality indicator because it is both readily available and independent of histology. Notably, a strength of using PDR-5 is that it includes both adenomas and sessile serrated lesions, and the latter may account for a substantial proportion of interval cancers [Bibr LI_LiteratureBookmark_30] . It should be noted that this study may not fully capture the effect of CADe on polyp detection when only polyps ≥5 mm are recorded. However, some criticism of CADe is that it only increases the detection of diminutive polyps with very low malignancy potential. Focusing on polyps ≥5 mm may thus reveal whether CADe affects the more relevant polyps. Future studies are needed to validate PDR-5 as a colonoscopy quality indicator.

Some inexperienced endoscopists performed relatively few endoscopies in phase 3, which may have led to underpowering of phase 3. This may have limited the ability to detect modest sustained effects of CADe. Future studies are needed to evaluate the learning effect of CADe among inexperienced endoscopists. As shown in previous studies and in the same region of Norway [Bibr LI_LiteratureBookmark_15] , patient age, sex, and colonoscopy indication significantly influence ADR/PDR-5. To adjust for this effect, we performed multivariable analyses. A possible limitation of the multivariable analyses is the risk of underpowering. We might have overlooked otherwise significant differences due to limited sample size. In particular, the inexperienced endoscopists had differences in the case-mix between phases 1 and 3, with higher age, more women, more symptoms, and fewer IBD patients in phase 3. This difference in case-mix may have influenced the PDR-5. Many of our patients in routine practice have the indications of symptoms and IBD, respectively. Other studies exclude patients with IBD, but we decided to include this indication as it reflects real-world data. However, it is a limitation that patients with IBD may exhibit different mucosal dysplasia features, which may not be detected by the training set used in GI Genius [Bibr LI_LiteratureBookmark_31] . Another limitation to the generalizability of this study is that the definition of an experienced endoscopist is not well established in the literature. Finally, we used only one type of AI device, which may limit the generalizability of our results. However, a study comparing CADe devices from different companies showed only minor differences between the producers [Bibr LI_LiteratureBookmark_32] .

Overall, these results support a targeted deployment strategy for CADe, prioritizing its use among trainees and early-career endoscopists, where a clear PDR-5 benefit is observed. The added value for high-volume, experienced endoscopists in real-world practice may be limited. Additionally, the absence of an observable post-exposure reduction in PDR-5 compared with pre-exposure argues against the idea that exposure to CADe erodes skills, at least for nonexperienced endoscopists, although such skill reduction has been observed in another study involving only experienced endoscopists [Bibr LI_LiteratureBookmark_10] .

### Conclusion

In this study, conducted in a real-world clinical setting, PDR-5 among inexperienced endoscopists increased during CADe use. In non-CADe-assisted colonoscopy, no upskilling or deskilling was observed after CADe exposure, regardless of the endoscopist experience level.

## Supplementary Material

Supplementary Material Zusatzmaterial
