# Supplementary material for: Learning and deskilling effects of artificial intelligence in colonoscopy among endoscopists with different levels of experience: a pragmatic, prospective trial
Source: Endoscopy. 2026 Jun 3;58(9):1003–14. doi: 10.1055/a-2858-7084 (PMC13552228; doi:10.1055/a-2858-7084)
Supplement: Supplementary file 1 [file 10-1055-a-2858-7084_28758089.pdf]

**Supplementary material**

# Learning and deskilling effects of artificial intelligence in colonoscopy among endoscopists with different levels of experience: a pragmatic, prospective trial

Tom Andre Pedersen, Yuichi Mori, Edoardo Botteri, Trond Engjom, Birgitte Seip, Georg Gjorgji Dimcevski, Roald Flesland Havre

**Table 1s** Patient and endoscopist characteristics, and quality indicators.

| | Total | Inexperienced | | | | Experienced | | | |
|---|---|---|---|---|---|---|---|---|---|
| | | Phase 1 | Phase 2 (CADe) | Phase 3 | *P* value | Phase 1 | Phase 2 (CADe) | Phase 3 | *P* value |
| Patients, n (%) | 5013 (100) | 678 (100) | 651 (100) | 476 (100) | 0.33 | 1075 (100) | 1088 (100) | 1045 (100) | 0.46 |
| Women | 2703 (53.9) | 343 (50.6) | 330 (50.7) | 260 (54.6) | | 609 (56.7) | 596 (54.8) | 565 (54.1) | |
| Men | 2310 (46.1) | 335 (49.4) | 321 (49.3) | 216 (45.4) | | 466 (43.3) | 492 (45.2) | 480 (45.9) | |
| Age, median (IQR), years | 59 (44–71) | 56 (40–71) | 54 (39–69) | 61 (43–73) | 0.10 | 59 (45–70) | 61 (46.25–71.75) | 59 (45–70) | 0.08 |
| Indications, n (%) | | | | | **<0.001** | | | | **0.03** |
| Symptoms | 3015 (60.1) | 353 (52.1) | 333 (51.2) | 300 (63.0) | | 658 (61.2) | 660 (60.7) | 711 (68.0) | |
| Surveillance (polyp, CRC) | 580 (11.6) | 71 (10.5) | 107 (16.4) | 69 (14.5) | | 123 (11.4) | 126 (11.6) | 84 (8.0) | |
| IBD | 664 (13.2) | 175 (25.8) | 163 (25.0) | 72 (15.1) | | 89 (8.3) | 87 (8.0) | 78 (7.5) | |
| CRC family/“screening”[1] | 262 (5.2) | 42 (6.2) | 8 (1.2) | 11 (2.3) | | 78 (7.3) | 69 (6.3) | 54 (5.2) | |
| Other | 458 (9.1) | 33 (4.9) | 35 (5.4) | 23 (4.8) | | 118 (11.0) | 138 (12.7) | 111 (10.6) | |
| Missing | 34 (0.7) | 4 (0.6) | 5 (0.8) | 1 (0.2) | | 9 (0.8) | 8 (0.7) | 7 (0.7) | |
| Inclusion time from first colonoscopy, mean (SD), months | 10.1 (10.0) | 6.8 (4.8) | 17.7 (7.3) | 23.8 (7.9) | | 2.2 (3.1) | 7.6 (7.7) | 11.8 (11.5) | |
| Age of endoscopists, mean (SD), years | | At study start: 41.1 years (4.6) | | | | At study start: 50.7 years (6.9) | | | |
| PDR-5, n/N (%) | 1525/5013 (30.4) | 216/678 (31.9) | 257/651 (39.5) | 173/476 (36.3) | **0.01** | 304/1075 (28.3) | 291/1088 (26.7) | 284/1045 (27.2) | 0.71 |
| Cecal intubation, n (%)[2] | | | | | 0.09 | | | | 0.83 |
| Yes | 4852 (97.8) | 634 (95.8) | 630 (97.4) | 462 (97.9) | | 1042 (98.3) | 1060 (98.4) | 1024 (98.1) | |
| No | 110 (2.2) | 28 (4.2) | 17 (2.6) | 10 (2.1) | | 18 (1.7) | 17 (1.6) | 20 (1.9) | |
| Missing | 51 | 16 | 4 | 4 | | 15 | 11 | 1 | |
| BBPS, n (%)[2] | | | | | 0.50 | | | | 0.27 |
| ≥6 | 4738 (95.0) | 619 (91.3) | 605 (92.9) | 441 (92.6) | | 1025 (95.9) | 1050 (97.1) | 998 (96.7) | |
| <6 | 249 (5.0) | 59 (8.7) | 46 (7.1) | 35 (7.4) | | 44 (4.1) | 31 (2.9) | 34 (3.3) | |
| Missing | 26 | 0 | 0 | 0 | | 6 | 7 | 13 | |
| Pain, severe, n (%)[2] | | | | | 0.69 | | | | 0.87 |
| Yes | 149 (6.4) | 18 (5.8) | 21 (7.2) | 14 (5.5) | | 33 (6.7) | 33 (6.7) | 30 (6.0) | |

| | | | | | | | | | |
|---|---|---|---|---|---|---|---|---|---|
| No | 2192 (93.6) | 290 (94.2) | 272 (92.8) | 239 (94.5) | | 461 (93.3) | 459 (93.3) | 471 (94.0) | |
| Missing | 2672 | 370 | 358 | 223 | | 581 | 596 | 544 | |
| Sedation/analgesia, n (%)[2] | | | | | 0.23 | | | | 0.44 |
| Yes | 2250 (45.4) | 395 (59.1) | 361 (56.2) | 255 (54.1) | | 417 (39.3) | 433 (40.1) | 389 (37.5) | |
| No | 2708 (54.6) | 273 (40.9) | 281 (43.8) | 216 (45.9) | | 643 (60.7) | 646 (59.9) | 649 (62.5) | |
| Missing | 55 | 10 | 9 | 5 | | 15 | 9 | 7 | |
| Withdrawal time[2,3] | | | | | **<0.001** | | | | **<0.001** |
| ≥6 minutes | 2140 (98.0) | 198 (95.2) | 234 (98.7) | 177 (94.7) | | 524 (99.2) | 553 (98.2) | 454 (98.7) | |
| <6 minutes | 43 (2.0) | 10 (4.8) | 3 (1.3) | 10 (5.3) | | 4 (0.8) | 10 (1.8) | 6 (1.3) | |
| Excluded | 2830 | 470 | 414 | 289 | | 547 | 525 | 585 | |
| Median (IQR) | 10 (7–12) | 10 (8–15) | 12 (9–20) | 9 (8–12) | | 9 (7–11) | 10 (7–11) | 9.5 (7–11) | |

**Bold** *P* values are significant.

BBPS, Boston Bowel Preparation Scale; CADe, computer-aided detection; CRC, colorectal cancer; IBD, inflammatory bowel disease; IQR, interquartile range; PDR-5, proportion of colonoscopies with ≥1 polyp ≥5 mm in size.

[1]"Screening" refers to surveillance colonoscopies in families with CRC or colonoscopies that are not part of the official CRC screening program.
[2]Percentages calculated based on available data.
[3]Excluded when biopsies were taken or intervention was performed.

**Table 2s** Proportion of colonoscopies with at least one polyp ≥5 mm in size (PDR-5) in phases 1 and 2.

| Variable/ category | All endoscopists | | | Inexperienced | | | Experienced | | |
|---|---|---|---|---|---|---|---|---|---|
| | PDR-5, n/N (%) | Univariable analysis | Multivariable analysis | PDR-5, n/N (%) | Univariable analysis | Multivariable analysis | PDR-5, n/N (%) | Univariable analysis | Multivariable analysis |
| | | OR (95%CI) | OR (95%CI) | | OR (95%CI) | OR (95%CI) | | OR (95%CI) | OR (95%CI) |
| Phase | | | | | | | | | |
| 1 (Before CADe) | 520/1753 (29.7) | Ref. | – | 216/678 (31.9) | Ref. | Ref. | 304/1075 (28.3) | Ref. | – |
| 2 (CADe) | 548/1739 (31.5) | 1.09 (0.94–1.26) | – | 257/651 (39.5) | 1.37 (1.09–1.72)[1] | 1.43 (1.11–1.84)[1] | 291/1088 (26.7) | 0.92 (0.76–1.12) | – |
| Sex | | | | | | | | | |
| Women | 515/1878 (27.4) | Ref. | Ref. | 207/673 (30.8) | Ref. | Ref. | 308/1205 (25.6) | Ref. | Ref. |
| Men | 553/1614 (34.3) | 1.39 (1.20–1.61)[1] | 1.33 (1.12–1.56)[1] | 266/656 (40.5) | 1.56 (1.24–1.97)[1] | 1.54 (1.20–1.98)[1] | 287/958 (30.0) | 1.28 (1.05–1.55)[1] | 1.14 (0.92–1.41) |
| Age | | | | | | | | | |
| 5-year increase | 1068/3492 (30.6) | 1.20 (1.17–1.23)[1] | 1.17 (1.14–1.20)[1] | 473/1329 (35.6) | 1.22 (1.18–1.27)[1] | 1.19 (1.15–1.24)[1] | 595/2163 (27.5) | 1.18 (1.14–1.22)[1] | 1.15 (1.11–1.19)[1] |
| Indication | | | | | | | | | |
| Symptoms | 595/2004 (29.7) | Ref. | Ref. | 240/686 (35.0) | Ref. | Ref. | 355/1318 (26.9) | Ref. | Ref. |
| Surveillance (polyp, CRC) | 241/427 (56.4) | 3.22 (2.58–4.02)[1] | 2.49 (1.97–3.14)[1] | 117/178 (65.7) | 3.73 (2.62–5.30)[1] | 2.69 (1.86–3.89)[1] | 124/249 (49.8) | 2.86 (2.15–3.82)[1] | 2.32 (1.71–3.13)[1] |
| IBD | 82/514 (16.0) | 0.42 (0.32–0.55)[1] | 0.52 (0.39–0.70)[1] | 71/338 (21.0) | 0.52 (0.38–0.71)[1] | 0.68 (0.49–0.95)[1] | 11/176 (6.3) | 0.21 (0.11–0.40)[1] | 0.25 (0.13–0.48)[1] |
| CRC family/"screening"[3] | 45/197 (22.8) | 0.84 (0.58–1.19) | 0.83 (0.58–1.20) | 18/50 (36.0) | 1.34 (0.72–2.47) | 1.36 (0.72–2.58) | 27/147 (18.4) | 0.66 (0.42–1.02)[2] | 0.70 (0.45–1.10) |
| Other | 98/324 (30.2) | 1.21 (0.93–1.58) | 1.02 (0.78–1.35) | 25/68 (36.8) | 1.26 (0.74–2.15) | 1.03 (0.59–1.79) | 73/256 (28.5) | 1.16 (0.85–1.58) | 1.00 (0.73–1.38) |
| Missing | 7/26 (26.9) | 0.80 (0.32–1.95) | 0.83 (0.33–2.12) | 2/9 (22.2) | 0.60 (0.12–2.97) | 0.58 (0.10–3.28) | 5/17 (29.4) | 0.85 (0.29–2.54) | 0.88 (0.29–2.71) |
| BBPS | | | | | | | | | |
| ≥6 | 1021/3299 (30.9) | Ref. | Ref. | 445/1224 (36.4) | Ref. | Ref. | 576/2075 (27.8) | Ref. | Ref. |

| | | | | | | | | | |
|---|---|---|---|---|---|---|---|---|---|
| <6 | 42/180 (23.3) | 0.62 (0.43–0.90)[1] | 0.56 (0.38–0.83)[1] | 28/105 (26.7) | 0.64 (0.40–1.01)[2] | 0.60 (0.37–0.97)[1] | 14/75 (18.7) | 0.59 (0.32–1.08)[2] | 0.54 (0.29–1.01)[2] |
| Missing | 13 | | | 0 | | | 13 | | |
| Cecal intubation | | | | | | | | | |
| Yes | 1037/3366 (30.8) | Ref. | – | 454/1264 (35.9) | Ref. | – | 583/2102 (27.7) | Ref. | – |
| No | 19/80 (23.8) | 0.66 (0.39–1.13) | – | 14/45 (31.1) | 0.82 (0.43–1.56) | – | 5/35 (14.3) | 0.45 (0.17–1.17) | – |
| Missing | 46 | | | 20 | | | 26 | | |
| Sedation/analgesia | | | | | | | | | |
| Yes | 443/1606 (27.6) | Ref. | Ref. | 255/756 (33.7) | Ref. | – | 188/850 (22.1) | Ref. | Ref. |
| No | 618/1843 (33.5) | 1.32 (1.13–1.55)[1] | 1.07 (0.90–1.27) | 213/554 (38.4) | 1.14 (0.90–1.44) | – | 405/1289 (31.4) | 1.52 (1.22–1.88)[1] | 1.39 (1.10–1.76)[1] |
| Missing | 43 | | | 19 | | | 24 | | |
| Inclusion time from first colonoscopy | | | | | | | | | |
| 3-month increase | 1068/3492 (30.6) | 1.02 (0.98–1.06) | – | 473/1329 (35.6) | 1.02 (0.97–1.07) | – | 595/2163 (27.5) | 0.99 (0.92–1.07) | – |
| Experience | | | | | | | | | |
| Experienced | 595/2163 (27.5) | Ref. | Ref. | – | – | – | – | – | – |
| Inexperienced | 473/1329 (35.6) | 1.56 (0.94–2.60)[2] | 1.86 (1.16–2.98)[1] | – | – | – | – | – | – |

The univariable analyses were based on generalized linear mixed models, with the endoscopist as a clustering random effect.

BBPS, Boston Bowel Preparation Scale; CADe, computer-aided detection; CIR, cecal intubation rate; CRC, colorectal cancer; IBD, inflammatory bowel disease; OR, odds ratio.

All univariable analyses with $P < 0.1$ were included in the multivariable analyses.
[1] $P < 0.05$.
[2] $P < 0.10$.
[3] "Screening" refers to surveillance colonoscopies in families with CRC or colonoscopies that are not part of the official CRC screening program.

**Table 3s** Proportion of colonoscopies with at least one polyp ≥5 mm in size (PDR-5) in phases 1 and 3.

| | All endoscopists | | | Inexperienced | | | Experienced | | |
|---|---|---|---|---|---|---|---|---|---|
| | PDR–5, n/N (%) | Univariable analysis | Multivariable analysis | PDR–5, n/N (%) | Univariable analysis | Multivariable analysis | PDR–5, n/N (%) | Univariable analysis | Multivariable analysis |
| Variable/Category | | OR (95%CI) | OR (95%CI) | | OR (95%CI) | OR (95%CI) | | OR (95%CI) | OR (95%CI) |
| Phase | | | | | | | | | |
| 1 (Before CADe) | 520/1753 (29.7) | Ref. | – | 216/678 (31.9) | Ref. | – | 304/1075 (28.3) | Ref. | – |
| 3 (After CADe) | 457/1521 (30.0) | 0.95 (0.82–1.11) | – | 173/476 (36.3) | 1.03 (0.79–1.34) | – | 284/1045 (27.2) | 0.93 (0.76–1.12) | – |
| Sex | | | | | | | | | |
| Women | 494/1777 (27.8) | Ref. | Ref. | 185/603 (30.7) | Ref. | Ref. | 309/1174 (26.3) | Ref. | Ref. |
| Men | 483/1497 (32.3) | 1.25 (1.08–1.46)[1] | 1.23 (1.04–1.45)[1] | 204/551 (37.0) | 1.35 (1.06–1.73)[1] | 1.35 (1.04–1.76)[1] | 279/946 (29.5) | 1.19 (0.98–1.45)[2] | 1.12 (0.90–1.38) |
| Age | | | | | | | | | |
| 5-year increase | 977/3274 (29.8) | 1.20 (1.17–1.23)[1] | 1.17 (1.14–1.20)[1] | 389/1154 (33.7) | 1.20 (1.16–1.25)[1] | 1.18 (1.13–1.22)[1] | 588/2120 (27.7) | 1.19 (1.15–1.23)[1] | 1.17 (1.13–1.21)[1] |
| Indications | | | | | | | | | |
| Symptoms | 574/2022 (28.4) | Ref. | Ref. | 217/653 (33.2) | Ref. | Ref. | 357/1369 (26.1) | Ref. | Ref. |
| Surveillance (polyp, CRC) | 190/347 (54.8) | 3.07 (2.42–3.90)[1] | 2.37 (1.85–3.03)[1] | 88/140 (62.9) | 3.44 (2.34–5.04)[1] | 2.74 (1.84–4.08)[1] | 102/207 (49.3) | 2.84 (2.09–3.87)[1] | 2.18 (1.58–2.99)[1] |
| IBD | 62/414 (15.0) | 0.44 (0.33–0.60)[1] | 0.56 (0.41–0.76)[1] | 47/247 (19.0) | 0.50 (0.35–0.72)[1] | 0.66 (0.45–0.98)[1] | 15/167 (9.0) | 0.33 (0.18–0.58)[1] | 0.38 (0.21–0.69)[1] |
| CRC family/"screening"[3] | 45/185 (24.3) | 0.92 (0.64–1.32) | 0.91 (0.63–1.31) | 17/53 (32.1) | 1.20 (0.65–2.22) | 1.19 (0.63–2.23) | 28/132 (21.2) | 0.79 (0.51–1.24) | 0.80 (0.51–1.26) |
| Other | 100/285 (35.1) | 1.51 (1.15–1.98)[1] | 1.29 (0.97–1.70) | 20/56 (35.7) | 1.25 (0.70–2.22) | 1.04 (0.57–1.90) | 80/229 (34.9) | 1.57 (1.56–2.14)[1] | 1.37 (1.00–1.88)[2] |
| Missing | 6/21 (28.6) | 0.97 (0.37–2.59) | 0.93 (0.34–2.55) | 0/5 (0.0) | – | – | 6/16 (37.5) | 1.41 (0.49–4.07) | 1.26 (0.42–3.73) |
| BBPS | | | | | | | | | |
| ≥6 | 928/3083 (30.1) | Ref. | – | 365/1060 (34.4) | Ref. | Ref. | 563/2023 (27.8) | Ref. | – |

| | | | | | | | | | |
|---|---|---|---|---|---|---|---|---|---|
| <6 | 45/172 (26.2) | 0.75 (0.52–1.07) | – | 24/94 (25.5) | 0.60 (0.37–0.98)[1] | 0.53 (0.32–0.90)[1] | 21/78 (26.9) | 0.98 (0.58–1.66) | – |
| Missing | 19 | | | 0 | | | 19 | | |
| Cecal intubation | | | | | | | | | |
| Yes | 946/3162 (29.9) | Ref. | – | 373/1096 (34.0) | Ref. | – | 573/2066 (27.7) | Ref. | – |
| No | 22/76 (28.9) | 0.93 (0.56–1.56) | – | 12/38 (31.6) | 0.85 (0.42–1.71) | – | 10/38 (26.3) | 1.05 (0.50–2.22) | – |
| Missing | 36 | | | 20 | | | 16 | | |
| Sedation/analgesia | | | | | | | | | |
| Yes | 407/1456 (28.0) | Ref. | Ref. | 210/650 (32.3) | Ref. | – | 197/806 (24.4) | Ref. | Ref. |
| No | 566/1781 (31.8) | 1.67 (0.99–1.37)[2] | 0.98 (0.82–1.18) | 175/489 (35.8) | 1.09 (0.84–1.40) | – | 391/1292 (30.3) | 1.24 (1.00–1.53)[2] | 1.15 (0.91–1.45) |
| Missing | 37 | | | 15 | | | 22 | | |
| Inclusion time from first colonoscopy | | | | | | | | | |
| 3-month increase | 977/3274 (29.8) | 0.99 (0.96–1.02) | – | 389/1154 (33.7) | 0.99 (0.95–1.03) | – | 588/2120 (27.7) | 0.99 (0.95–1.03) | – |
| Experience | | | | | | | | | |
| Experienced | 588/2120 (27.7) | Ref. | – | – | – | – | – | – | – |
| Inexperienced | 389/1154 (33.7) | 1.37 (0.80–2.34) | – | – | – | – | – | – | – |

The univariable analyses were based on generalized linear mixed models, with the endoscopist as a clustering random effect.

BBPS, Boston Bowel Preparation Scale; CADe, computer-aided detection; CIR, cecal intubation rate; CRC, colorectal cancer; IBD, inflammatory bowel disease; OR, odds ratio.

All univariable analyses with $P < 0.1$ were included in the multivariable analyses.
[1] $P < 0.05$.
[2] $P < 0.10$.
[3] "Screening" refers to surveillance colonoscopies in families with CRC or colonoscopies that are not part of the official CRC screening program.
